# Effects of Different Crop Rotations on Microbial Diversity and Enzyme Activities in *Brassica napus* Rhizosphere Soil

**DOI:** 10.3390/microorganisms14010091

**Published:** 2025-12-31

**Authors:** Xiaona Tian, Jia Duan, Hongli Huo, Jiuru Huangfu, Mengjiao Yan, Huilin Lu, Ziqin Li, Peiling Song

**Affiliations:** Plant Protection Institute, Inner Mongolia Academy of Agricultural and Animal Husbandry Sciences, Hohhot 010031, China; 18447053580@163.com (X.T.);

**Keywords:** *Brassica napus*, preceding crop, microbial community, enzyme activity, physicochemical properties, functional prediction

## Abstract

Continuous cropping of *Brassica napus* impairs sustainable production via soil nutrient imbalance and microecological degradation. We evaluated rhizosphere soil properties and microbial communities under rotations crops (*Triticum aestivum* [TaBn], *Beta vulgaris* [BvBn], *Glycine max* [GmBn], *Sorghum bicolor* [SbBn], *Hordeum vulgare* [HvBn], and *Brassica napus* [BnBn]). BvBn had the highest total nitrogen, total potassium, available potassium, and organic matter contents. TaBn exhibited the highest soil enzyme activities, and its bacterial/fungal Chao1/Simpson indices and unique operational taxonomic units (OTUs; bacteria: 333, fungi: 37) exceeded other patterns. Principal coordinate analysis showed distinct microbial community separation in BvBn/TaBn versus BnBn. TaBn enriched dominant bacterial phyla Pseudomonadota and Actinomycetota; all preceding crops increased fungal phylum Ascomycota while reducing Mucoromycota. Comprehensive assessment confirmed all preceding crops, except oilseed rape altered rhizosphere microbial structure, with TaBn as the optimal preceding crops.

## 1. Introduction

Rapeseed (*Brassica napus*) is widely cultivated in China, characterized by a large planting area, high yield, and significant economic benefits, making it crucial for ensuring the national edible oil security [1,2,3]. However, during its growth period, rapeseed is susceptible to diseases such as sclerotinia and downy mildew. Additionally, continuous cropping can disrupt the soil microecological balance (leading to an imbalance between beneficial and harmful microorganisms, resulting in a surge of pathogenic bacteria) and cause soil nutrient imbalance (deficiencies in elements such as potassium and boron, along with excessive accumulation of nitrogen and phosphorus). These issues collectively lead to a decline in both the yield and quality of rapeseed, causing economic losses for farmers [4].

Traditional pest and disease control has largely relied on chemical pesticides, which, while alleviating issues to some extent, can lead to negative consequences, such as pesticide residues, environmental pollution, and increased pest resistance, due to long-term use. In contrast, crop rotation, as an ecologically friendly cultivation practice, can effectively regulate pests and diseases, improve soil structure, and enhance land utilization by adjusting the planting sequence and combinations of different crops [5]. It is a key approach to alleviating continuous cropping obstacles and maximizing the benefits of rapeseed cultivation. Existing studies have shown that different rotation patterns can alter the physical and chemical properties of the soil, microbial abundance, and enzyme activity, thereby affecting the metabolic capacity and diversity of soil microbial communities [6,7]. For instance, under varying fertilization conditions, the retention of soil organic nitrogen in the rapeseed-rice rotation model surpasses that of the wheat-rice rotation. Significant differences in soil nitrogen content are observed following rotation and continuous cropping treatments, with crop rotation enhancing nitrogen levels in the soil and, consequently, improving soil fertility [8]. Research indicates that crop rotation can also enhance the effective phosphorus content in the soil [9,10]. Potassium deficiency poses a challenge to continuous cropping [11], as the availability of readily accessible potassium in the soil significantly influences crop growth and development. In agricultural production, insufficient soil potassium levels often result in diminished crop yields. Implementing diverse crop rotations can effectively mitigate this issue; for instance, the potato–double rice rotation helps maintain soil potassium content at a relatively stable level [12]. Furthermore, there are differences in the characteristics of soil microbial communities under continuous rapeseed cropping in different regions, such as the dominance of Flavobacterium and actinomycetes (especially the genus Streptomyces) in certain areas, while various pathogenic genera are commonly found in the fungal community [13].

Based on the high-altitude and cold climatic characteristics of Hulunbuir City in Inner Mongolia, a field experiment was conducted to investigate the effects of six preceding crops on rhizosphere microorganisms in rapeseed soil, under a one-crop-per-year condition. The preceding crops included *Triticum aestivum*, *Beta vulgaris*, *Glycine max*, *Sorghum bicolor*, and *Hordeum vulgare*.

Currently, data regarding the changes in soil microorganisms associated with the rotation of rapeseed and different preceding crops is relatively scarce, making this study distinctly innovative. Given that selecting the optimal rapeseed preceding crop scheme requires the evaluation of numerous preceding crop combinations, conducting short-term preliminary experiments is an efficient and feasible research approach. Furthermore, directly measuring crop yield in short-term experiments poses significant challenges, while changes in soil microbial communities can serve as reliable indicators reflecting soil nutrient status. Accordingly, this study proposes the following research objectives and hypotheses: Objective: To clarify the impact of different preceding crops on soil microbial diversity by analyzing the enzyme activity, physicochemical properties, and metagenomic characteristics of the rhizosphere soil of rapeseed under six patterns (including a continuous cropping control). Hypothesis: compared to continuous cropping, a reasonable preceding crop can significantly improve the soil microecological environment and nutrient status, and there are differences in the regulatory effects of different preceding crops. This study aims to lay the foundation for selecting the optimal rapeseed preceding crop combinations suitable for the Hulunbuir region.

## 2. Materials and Methods

### 2.1. Overview of the Experimental Site

The experiment was conducted from May to September in 2023 and 2024 at the experimental station of the Hulunbuir Agricultural Reclamation Te Ni River Farm Co., Ltd., Hulunbuir, China, located at 120°23′ E longitude and 49°31′ N latitude, with an elevation of 680 m. The soil in the experimental site is chernozem soil. The area has a temperate continental monsoon climate. The physical and chemical characteristics of the tillage layer soil were organic matter of 43.6 g kg^−1^; total nitrogen of 2.27 g kg^−1^; available phosphorus of 35.0 mg kg^−1^; and available potassium of 173 mg kg^−1^. The cultivation area of the different rotation patterns was about 2 hm^2^. *Triticum aestivum*, *Beta vulgaris*, *Glycine max*, *Sorghum bicolor*, *Hordeum vulgare,* and *Brassica napus* were planted in 2023, and rapeseed was planted after different preceding crops in 2024.

### 2.2. Experimental Design

During the maturation period of rapeseed, an observational method and a ‘W’-shaped, five-point sampling method were employed, with each sampling plot covering an area of 30 m^2^ (5 m × 6 m). Ten rapeseed plants were randomly selected from each plot, ensuring that the selected target plants were spaced at least 1 m apart, resulting in a total of 50 plants. The selected Brassica napus were gently uprooted, and the method of root shaking was utilized to obtain rhizosphere soil under different cropping patterns [14]. Six cropping patterns were established: *Brassica napus-Brassica napus* (BnBn), *Triticum aestivum-Brassica napus* (TaBn), *Beta vulgaris-Brassica napus* (BvBn), *Glycine max-Brassica napus* (GmBn), *Sorghum bicolor-Brassica napus* (SbBn), and *Hordeum vulgare-Brassica napus* (HvBn). Replicate BnBn, TaBn, BvBn, GmBn, SbBn, and HvBn means six independent experiments, with each experiment containing three technical replicate values. BnBn served as the control group for comparative analysis with other rotations. The soil samples were thoroughly mixed, and residual roots and impurities were removed in preparation for subsequent enzyme activity determination and microbial community analysis in the soil. The experiment lasted for two years, with six different crops planted in 2023 and all fields planted with rapeseed in 2024.

### 2.3. Determination of Soil Enzyme Activity

To determine enzyme activity, soil samples must be air-dried and ground, with the dry weight of the soil serving as the basis for measurement. Various kits were employed to measure soil urease (S-UE), soil sucrase/acid invertase (S-SC), soil acid phosphatase (S-ACP), soil acid protease (S-ACPT), soil β-glucosidase (S-β-GC or S-GC), and soil catalase (S-CAT). The corresponding kit numbers are as follows: S-UE (G0301F), S-SC (G0302F), S-ACP (G0304F), S-ACPT (G0316F), S-β-GC (G0312F), and S-CAT (G0303F). All kits were procured from Suzhou Greys Biotechnology Co., Ltd. (Suzhou, China). The enzyme activity of the samples was assessed using a microplate method with a microplate reader (SpectraMax iD3, Molecular Devices, Shanghai, China).

### 2.4. Determination of Soil Physicochemical Properties

The pH of the soil was measured using potentiometric methods. Total nitrogen content in the soil was determined using the Kjeldahl method, while total potassium was measured through flame photometry following fusion with NaOH. Total phosphorus was quantified using the molybdenum-antimony colorimetric method after NaOH fusion. Available potassium in the soil was extracted with 1 mol·L^−1^ NH_4_OAc and measured by flame photometry. Soil organic matter was assessed using the potassium dichromate–concentrated sulfuric acid method, which included heat treatment. Available phosphorus was determined using the molybdenum–antimony colorimetric method after extraction with 0.5 mol·L^−1^ NaHCO_3_ [15,16].

### 2.5. Metagenomic Sequencing

DNA was extracted from rhizosphere soil samples subjected to various preceding crops (TIANGEN, DP712-02). The qualified DNA was purified and recovered using the TruSeq library preparation kit magnetic beads, followed by fragmentation. The genomic library was constructed from the fragmented products using the TruSeq Nano DNA LT Library Preparation Kit (Illumina, FC-121-4001, Shanghai, China). The library was quantified utilizing Qubit 1X dsDNA HS Assay Kits (Invitrogen, Q33230, Shanghai, China). Finally, paired-end sequencing was conducted on the Illumina Novaseq 6000 following standard procedures, employing a sequencing mode of PE150 and utilizing the NovaSeq 6000 XP 4-Lane Kit v1.5 (300 cycles) (Illumina, 20043131). Subsequent to sequencing, the data were analyzed using the Microbial Alliance Analysis Platform based on Reads (reference-based) metagenomics.

### 2.6. Data Analysis

Using Kraken2 (2.0.7-beta) [17], the sequence data from each sample, following quality control and host removal, were compared against a self-constructed microbial nucleic acid database to quantify the number of sequences corresponding to the species present in the samples. After classifying the metagenomic sequencing data with Kraken, Bracken 2.0 [18] was employed, utilizing default parameters, to perform a Bayesian re-estimation of abundance based on the classification results obtained from Kraken2, thereby estimating the species-level abundance of the metagenomic samples. The analyses of canonical correspondence analysis (CCA) and redundancy analysis (RDA) primarily relied on the R language package vegan (2.6.0), with visualizations created using ggplot2 (3.3.5.9000) [19]. We used PERMANOVA to test the significant effects of different treatments on the community structure of bacteria and fungi. Additionally, we mapped the differences between samples using three methods: PCA, PCoA, and NMDS, based on the Bray–Curtis distance matrix.

The information gathered for this study was subjected to thorough statistical analysis, utilizing Microsoft Excel 2021 alongside IBM SPSS Statistics 26. Various differential analysis methods, such as LEfSe 1.0.8 [20] (linear discriminant analysis effect size), Kruskal–Wallis and Dunn’s Test (using the R package dunn.test 1.3.5 [21]), ANOVA, and Duncan’s test (using the R package ggpubr 0.2.4 [22]), were employed to compare the differences in species and functions between groups.

## 3. Results

### 3.1. The Effects of Different Preceding Crop on Rapeseed Soil Physicochemical Properties and Enzyme Activities

The physicochemical properties of the soil were assessed under various preceding crops (Table 1). Notably, the pH level in the HvBn was 2.5% higher than that in the BnBn. Additionally, the BvBn exhibited significant increases in total nitrogen (TN), total potassium (TK), available potassium (AK), and organic matter (OM) compared to BnBn, with increases of 13.71%, 10.45%, 27.65%, and 11.12%, respectively. The AK content in the SbBn and TaBn was also higher than in the BnBn, by 10.25% and 18.97%, respectively.

**Figure 1:**
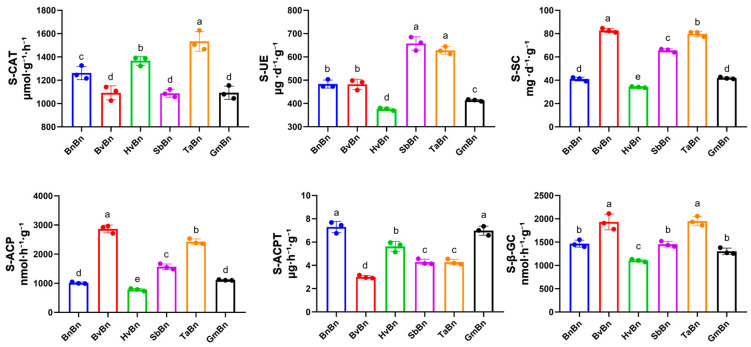
The effects of different preceding crops on soil enzyme activities. The letters a, b, c, d and e indicate statistically significant differences, *p* < 0.05 (a > b > c > d > e).

Enzyme activity assessments were conducted on soils subjected to various preceding crops (Figure 1). Compared to the BnBn, rotating with *Glycine max* had the most pronounced effect on S_CAT and S_UE enzyme activities, leading to reductions of 13% and 15%, respectively. Interestingly, rotating *Hordeum vulgare* increased S_CAT enzyme activity by 8.3%, while rotating *Sorghum bicolor* enhanced S_UE, S_ACP, and S_SC activities by 36.4%, 54.8%, and 60%, respectively. Furthermore, rotation with *Beta vulgaris* resulted in significant increases in S_ACP, S_GC, and S_SC activities, by 183%, 31.7%, and 101.7%, respectively. Rotation with *Triticum aestivum* enhanced all five enzyme activities, excluding S_ACPT, with increases of 21.5%, 29.9%, 139.7%, 32.9%, and 94.6% for S_CAT, S_UE, S_ACP, S_GC, and S_SC, respectively.

These results suggest that the activities of S_ACP, S_GC, and S_SC remained largely stable or elevated following various patterns. However, rotations involving *Beta vulgaris*, *Hordeum vulgare*, *Sorghum bicolor*, *Glycine max*, and *Triticum aestivum* did not enhance the activity of S_ACPT, with the lowest S_ACPT activity observed after *Beta vulgaris* rotation, showing a decrease of 58.8%.

### 3.2. Statistical Analysis of OTUs Under Different Preceding Crops

**Figure 2:**
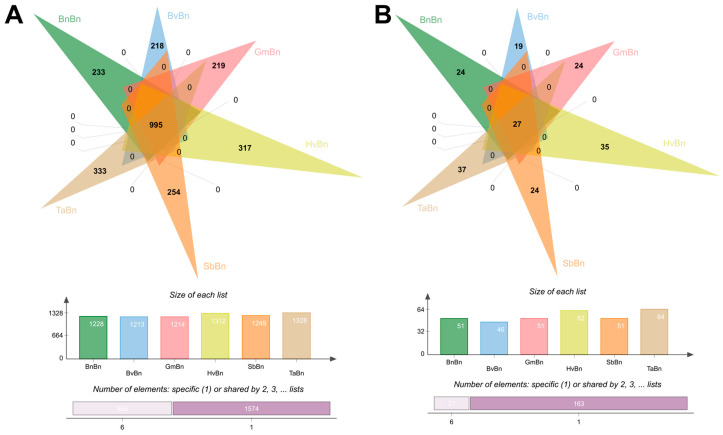
OTUs of (**A**) bacteria and (**B**) fungi under different preceding crops.

In this study, a total of 2569 bacterial operational taxonomic units (OTUs) were identified across six patterns. Among these, 995 OTUs were shared across all systems, representing 38.73% of the total OTUs (Figure 2A). The monoculture system BnBn yielded 233 OTUs, accounting for 9% of the total, while the TaBn system exhibited the highest bacterial diversity with 333 OTUs, constituting 13% of the total. The remaining systems—BvBn, GmBn, SbBn, and HvBn—contained 218 (8.5%), 219 (8.5%), 254 (9.9%), and 317 (12.3%) OTUs, respectively. In terms of fungal communities, a total of 190 OTUs were detected across all preceding crops, of which 27 OTUs were common (Figure 2B). The TaBn system again demonstrated the highest fungal diversity, comprising 37 OTUs, which represents 19.5% of the total. Conversely, the BvBn system exhibited the lowest fungal diversity, with only 19 OTUs, accounting for 10% of the total.

### 3.3. Alpha-Diversity Indices Analysis

**Figure 3:**
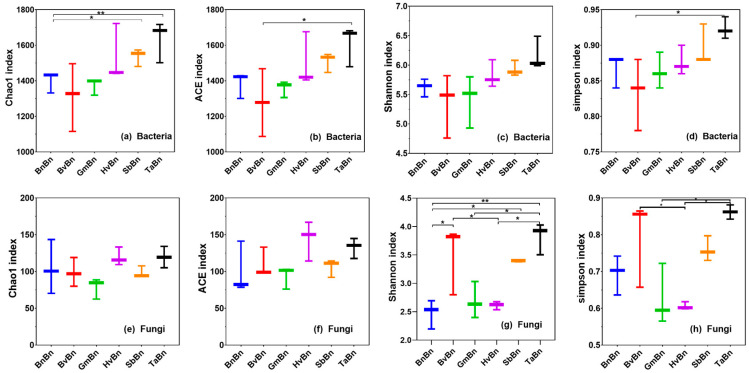
Microbial diversity and abundance in different treatments. Chao1 (**a**), ACE (**b**), Shannon (**c**), and Simpson (**d**) are indices of alpha diversity in bacteria; Chao1 (**e**), ACE (**f**), Shannon (**g**), and Simpson (**h**) are indices of alpha diversity in fungi. Data were analyzed utilizing Microsoft Excel 2021, alongside IBM SPSS Statistics 26. * *p* = 0.05 and ** *p* = 0.01.

The rarefaction curves showed that all samples reached saturation at approximately 2 × 10^5^ sequences, indicating sufficient sequencing depth to capture the majority of microbial diversity (Supplementary Figure S1). Alpha diversity analysis revealed divergent patterns between bacterial and fungal communities across different treatments (Figure 3). In the bacterial community, BnBn had a significantly lower Chao1 index than SbBn (*p* = 0.008) and TaBn (*p* = 0.048), while TaBn exhibited higher ACE (*p* = 0.026) and Simpson indices (*p* = 0.042) than BvBn. For fungi, TaBn showed the highest Simpson index (significant vs. GmBn, *p* = 0.008; vs. HvBn, *p* = 0.004) and Shannon index (significant vs. BnBn, GmBn, and HvBn), whereas BnBn had the lowest Shannon index. No significant differences in Chao1 and ACE indices were detected among the six fungal community groups.

### 3.4. Beta-Diversity Indices Analysis

**Figure 4:**
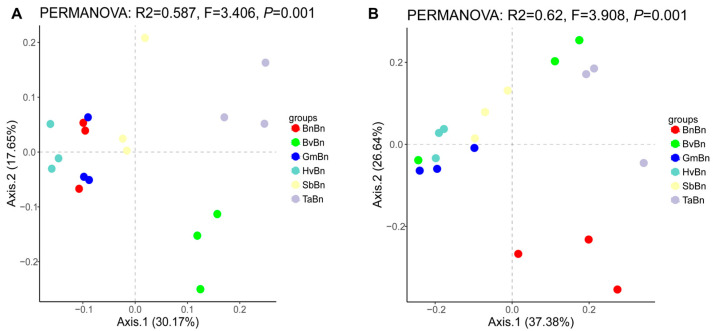
Principal component analysis (PCoA) of bacteria (**A**) and fungi (**B**) under different preceding crops. The horizontal axis denotes the first principal coordinate, with the percentage shown in parentheses indicating the contribution rate of this coordinate to the variation among samples; the vertical axis indicates the second principal coordinate, and the percentage in parentheses reflects the contribution rate of the second principal coordinate to the sample variation.

At the OTU level, PCoA revealed that the first two Axis1 and Axis2 accounted for 30.17% and 17.65% of the total variation in soil bacteria, respectively (Figure 4A). The PCoA plot of the bacterial community structure indicated that the BvBn and TaBn groups exhibited distinct community structures, positioned in the bottom-right and top-right quadrants, respectively. In contrast, the BnBn and GmBn groups displayed similar community structures, clustering on the left side of the plot. The HvBn group was relatively independent, while the SbBn group was situated closer to the origin, suggesting some overlap with other groups. The results of the PCoA analysis indicate that different preceding crops have a significant impact on the microbial community structure, with samples from different groups showing distinct separation on the ordination plot. This finding is further corroborated by the PERMANOVA analysis (PERMANOVA: F = 3.406, *p* = 0.001), which reveals a statistically significant difference.

The contribution rates of soil fungi were 37.38% and 26.64%, respectively (Figure 4B). The BnBn group, located in the lower right corner, demonstrated an independent and distinct pattern compared to other groups, with significant differences in fungal communities (PERMANOVA: F = 3.908, *p* = 0.001).

### 3.5. Relative Abundance of Bacteria and Fungi

**Figure 5:**
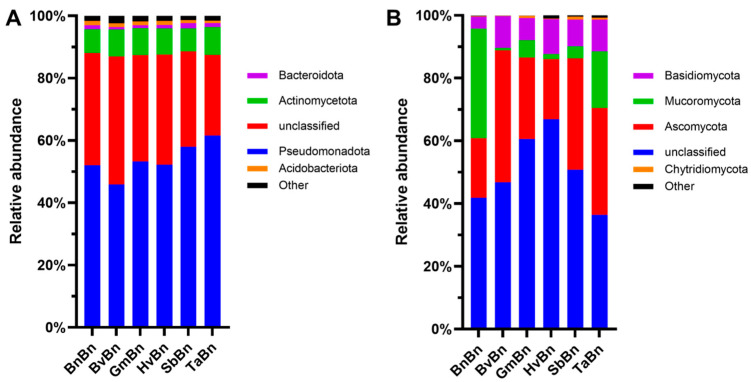
Abundance of bacterial (**A**) and fungal (**B**) phyla under different preceding crops.

Across all six patterns, Pseudomonadota was the overwhelmingly dominant phylum, accounting for 45.8–61.6% of the bacterial community (Figure 5A); its relative abundance peaked under TaBn (61.6%) and reached its minimum under BvBn (45.9%). Unclassified bacteria constituted the second-largest fraction, ranging from 25.9% to 41.1%, with the highest value recorded in BvBn (41.1%) and the lowest in TaBn (26.0%). Actinomycetota showed little variation among treatments, fluctuating narrowly between 7.4% and 8.8%. The Bacteroidota and Acidobacteriota were present only in minor proportions (<2%); Bacteroidota attained its greatest abundance under SbBn (1.7%), whereas Acidobacteriota declined to its lowest level under TaBn (0.8%). The relative abundance of soil fungal phylum differed markedly among the six patterns (Figure 5B).

In every treatment, unclassified fungi accounted for the largest share (36.4–66.9%). Beyond this dominant group, the BnBn regime was characterized by a high proportion of Mucoromycota (34.9%), whereas Ascomycota and Basidiomycota remained comparatively low (19.1% and 3.8%, respectively). Shifting to BvBn elevated Ascomycota to 42.1%, making it the second most abundant group. Under GmBn, Ascomycota declined to 26.0% and Mucoromycota dropped to only 5.5%. In the HvBn, Ascomycota remained near the BnBn level (19.2%), but Basidiomycota increased noticeably to 11.1%. The SbBn raised Ascomycota to 35.5%, while TaBn maintained both Ascomycota (34.1%) and Mucoromycota (18.1%) at relatively high levels.

**Figure 6:**
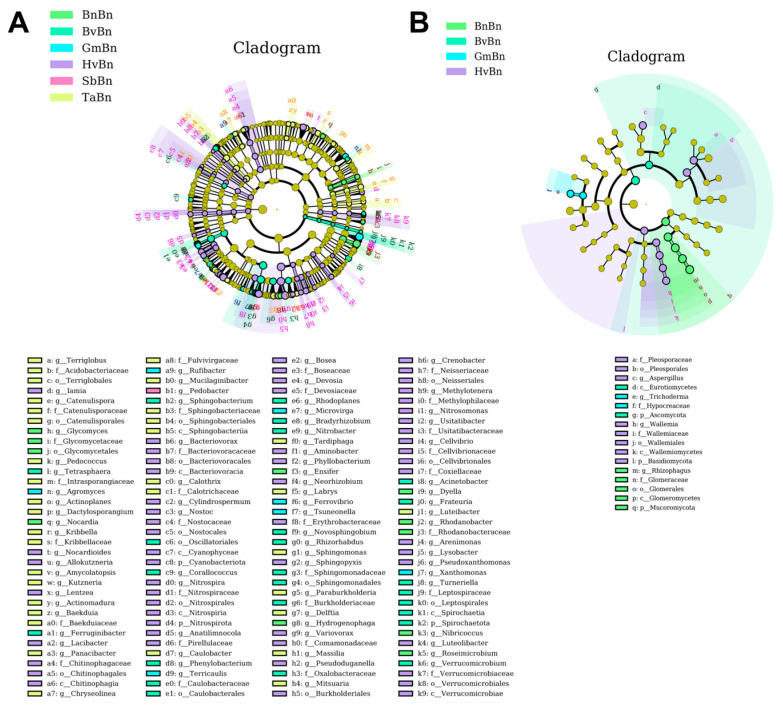
LEfSe analysis of bacteria (**A**) and fungi (**B**) under different patterns. The cladogram is structured from the innermost to the outermost levels, corresponding to different taxonomic ranks such as domain, phylum, class, order, family, genus, and species. The connecting lines between the levels represent the relationships of belonging. Each circular node represents a microorganism, with yellow nodes indicating that the differences among groups are not significant. Nodes that are not yellow signify that the microorganism is a characteristic species of the corresponding color group (with significantly higher abundance in that group). The colored sector areas denote the subordinate taxonomic intervals of the characteristic microorganisms.

LEfSe analysis was employed to identify indicator taxa associated with abundance changes in different preceding crop, utilizing a threshold of LDA score > 2 and *p* < 0.05. A total of 136 taxa, ranging from phylum to genus, were identified in bacteria, alongside 17 in fungi. The counts of bacterial biomarkers in the BnBn, BvBn, GmBn, HvBn, SbBn, and TaBn groups were 10, 26, 7, 57, 1, and 35, respectively (Figure 6A). In contrast, the fungal counts in the same groups were 4, 2, 2, 8, 0, and 0, respectively (Figure 6B). The bacterial biomarkers were predominantly distributed across Acidobacteriota, Actinomycetota, Bacteroidota, Bdellovibrionota, Cyanobacteriota, Myxococcota, Nitrospirota, Planctomycetota, Pseudomonadota, Spirochaetota, and Verrucomicrobiota. Fungal biomarkers were primarily found within Ascomycota, Basidiomycota, and 65 Mucoromycota species.

We identified the indicator taxa for each preceding crop at the genus level, noting that the differences in biomarkers were primarily influenced by the crop type. The indicator genera for BnBn included *Glycomyces*, *Nocardia*, *Ensifer*, *Hydrogenophaga*, *Dyella*, *Rhodanobacter*, et al. For BvBn, the genera were *Tetrasphaera*, *Ferruginibacter*, *Sphingobacterium*, *Corallococcus*, *Phenylobacterium*, *Rhodoplanes*, et al. In the case of GmBn, the indicator genera comprised *Agromyces*, *Rufibacter*, *Terricaulis*, *Microvirga*, *Ferrovibrio*, *Tsuneonella*, and *Xanthomonas.* The endemic genera for HvBn included *Iamia*, *Nocardioides*, *Allokutzneria*, *Lentzea*, *Lacibacter*, *Bacteriovorax*, *Cylindrospermum*, et al. The endemic genus for SbBn was *Pedobacter*, while TaBn included *Terriglobus*, *Catenulispora*, *Pedococcus*, *Actinoplane*, *Dactylosporangium*, *Kribbella*, et al. Notably, TaBn, SbBn, and BvBn did not exhibit any endemic genera at the fungal level. The endemic genera for HvBn were *Aspergillus* and *Wallemia*, while *Rhizophagus* was identified for BnBn, and *Trichoderma* for GmBn.

### 3.6. Soil Drivers of Microbial Community Change

**Figure 7:**
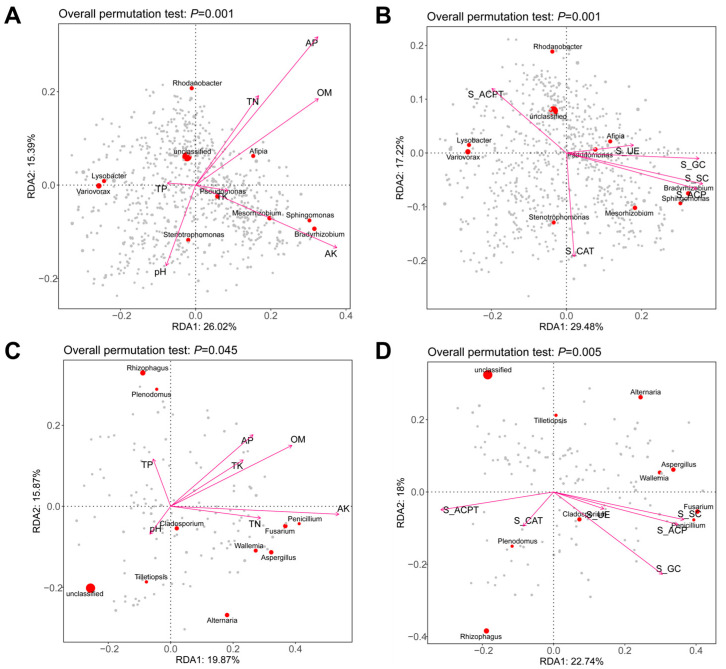
Environmental drivers of soil microbial community composition. Redundancy analysis (RDA) was performed on individual sample bacteria with soil physicochemical properties (**A**) and enzyme activity (**B**), as well as on fungal communities with soil physicochemical properties (**C**) and enzyme activity (**D**). The direction of the arrows indicates the correlation with the first two canonical axes, while the length of the arrows represents the strength of the correlation.

The RDA ordination plot revealed a significant correlation between microbial communities and environmental variables (overall permutation test: *p* = 0.001). Environmental variables such as AP, OM, TN, and AK showed strong positive associations with bacterial genera including *Rhodanobacter*, *Pseudomonas*, *Mesorhizobium*, and *Bradyrhizobium*. In contrast, *Lysobacter* and *Variotax* were closely associated with lower pH and higher TP levels (Figure 7A). *Rhodanobacter* and *Pseudomonas* were positively associated with S_ACPT and S_UE, whereas *Stenotrophomonas* was negatively correlated with S_CAT. *Mesorhizobium* clustered near S_ACPT and S_SC, indicating potential adaptation to these conditions (Figure 7B).

In fungi, OM and AK showed strong positive correlations with RDA1, driving the abundance of *Penicillium* and *Aspergillus*. TP negatively correlated with RDA1, while pH negatively associated with RDA2 and was linked to *Cladosporium* distribution. *Tilletiopsis* exhibited a negative response to TN. Key environmental factors, including S_ACP (positively correlated with *Aspergillus* and *Penicillium*) and S_ACPT, significantly structured the microbial community (*p* = 0.005). Features such as Cladosporium and *Alternaria* showed strong associations with S_ACP, while *Rhizophagus* was linked to S_GC (Figure 7D).

**Figure 8:**
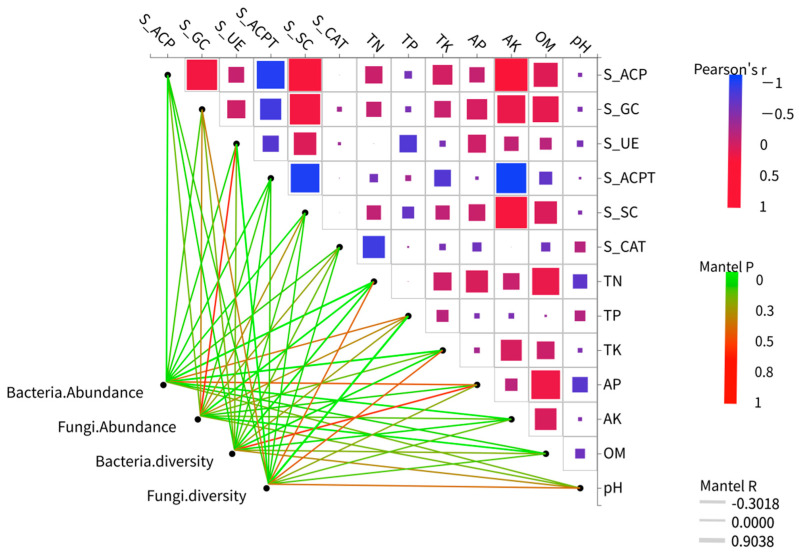
Heatmaps of soil factors and microbial diversity. The Mantel test was employed to determine the correlation between the species richness and diversity of bacterial and fungal communities and soil properties, as well as enzyme activity. The Mantel R reflects the strength of the distance correlation between the two matrices, while the Mantel P tests the significance of this correlation coefficient.

The effects of various environmental factors on the diversity of soil bacterial and fungal communities were assessed using the Mantel test. TN significantly influenced bacterial diversity (r^2^ = 0.2707, *p* = 0.049). Additionally, TN (r^2^ = 0.3766, *p* = 0.001), total potassium (TK) (r^2^ = 0.3283, *p* = 0.023), AK (r^2^ = 0.2458, *p* = 0.032), and S_ACP (r^2^ = 0.2274, *p* = 0.034) all significantly impacted bacterial abundance. Furthermore, TN (r^2^ = 0.2732, *p* = 0.026) and OM (r^2^ = 0.2456, *p* = 0.027) significantly affected fungal abundance, while S_UE (r^2^ = 0.2659, *p* = 0.01) and TP (r^2^ = 0.4948, *p* = 0.002) significantly influenced fungal diversity (Figure 8).

### 3.7. Functional Prediction of Root-Associated Fungal Communities

**Figure 9:**
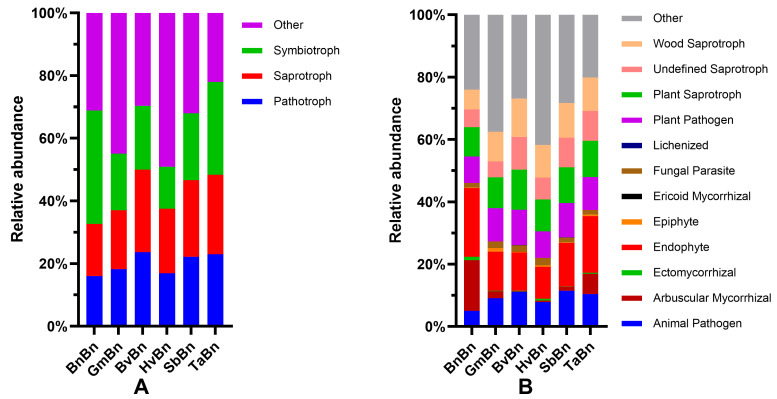
FUNGuild Functional Classification of Fungal Communities: (**A**) classification of fungal trophic types; (**B**) fungal guild functional classification.

Based on the FUNGuild database, we conducted a preliminary prediction of the fungal community, comparing three abundant trophic types and twelve common ecological functional groups (Figure 9A). The relative abundance of Symbiotroph fungi ranges from approximately 13% to 36%. Among the various cropping systems, the highest relative abundance of symbiotic trophic fungi is observed in BnBn at 36.12%, while the lowest is noted in HvBn at 13.43%. The relative abundances for the other cropping systems are as follows: GmBn 18.02%, BvBn 20.4%, SbBn 21.32%, and TaBn 29.64%. The relative abundance of Saprotroph fungi ranges from approximately 16% to 25%. The highest relative abundance of saprophytic trophic fungi is found in TaBn at 25.34%, whereas the lowest is recorded in BnBn at 16.62%. The relative abundances for the other cropping systems include: GmBn 18.80%, BvBn 26.38%, HvBn 20.62%, and SbBn 26.38%. The relative abundance of Pathotroph fungi ranges from approximately 16% to 23%. The highest relative abundance of pathogenic trophic fungi is observed in BvBn at 23.60%, while the lowest is found in BnBn at 16.06%. The relative abundances for the other cropping systems are as follows: GmBn 18.24%, HvBn 16.89%, SbBn 22.18%, and TaBn 23.02%.

An analysis of twelve common ecological functional groups under various preceding crops indicates that both Endophyte and Plant Saprotroph consistently rank among the top three groups across the six preceding crops examined (Figure 9B). The relative abundance of endophytic fungi fluctuates between approximately 10% and 22%, while the relative abundance of plant saprotrophs ranges from 9% to 13%. Furthermore, in the BnBn patterns, the relative abundance of *Arbuscular Mycorrhizal* is notably high at 16.23%. In the GmBn patterns, the relative abundance of Plant Pathogen is elevated at 10.69%. The relative abundances of *Wood Saprotroph* in the BvBn, TaBn, and HvBn patterns are recorded at 12.28%, 10.73%, and 10.46%, respectively. Lastly, the SbBn patterns exhibit a relatively high abundance of Animal Pathogen at 11.49%.

### 3.8. Functional Prediction of Root-Associated Bacteria Communities

**Figure 10:**
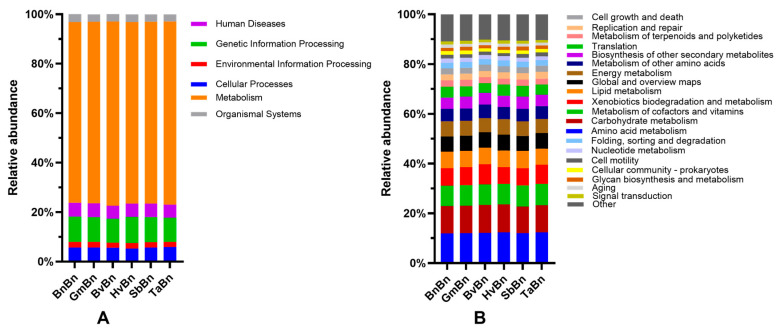
Abundance of the functional genes of soil bacteria. (**A**): Primary metabolic pathways in bacteria; (**B**): Secondary metabolic pathways in bacteria.

In the KEGG PATHWAY database, biological metabolic pathways are categorized into six primary categories: Human Diseases, Genetic Information Processing, Environmental Information Processing, Cellular Processes, Metabolism, and Organismal Systems (Figure 10A). The relative abundance of three functional accumulations—metabolism (ranging from 72.97% to 74.43%), genetic information processing (from 9.69% to 10.53%), and cellular processes (from 5.27% to 5.73%)—exceeds 89%. Notably, there are no significant differences in the relative abundance of these functions across different cropping treatment modes. In terms of secondary functional predictions, over 89% of the differential genes in soil bacteria are concentrated within the top 20 pathways, with 14 functional genes exhibiting an average relative abundance exceeding 2% (Figure 10B). The top three categories in terms of relative abundance are Amino Acid Metabolism (11.97% to 12.37%), Carbohydrate Metabolism (10.73% to 11.24%), and Metabolism of Cofactors and Vitamins (8.18% to 8.58%). However, similar to first-level functional predictions, there are no significant differences in the relative abundance of these functions among various cropping treatment modes.

## 4. Discussion

Inner Mongolia cultivates nearly 4 million mu of spring rapeseed, making it the largest production area for spring rapeseed in China, with over 80% of the cultivation concentrated in the Hulunbuir region. Affected by the region and climate, the region is ripe once a year, and the crops that can be planted are limited. The purpose of this study was to identify the most suitable crop for rotation with rapeseed.

### 4.1. Synergistic Changes in Soil Chemical and Biological Fertility

Different preceding crops shape distinct soil microenvironments through their unique root exudates and residues. In this study, BvBn exhibited the most remarkable performance in terms of chemical fertility; as a deep-rooted crop, it can significantly increase the total nitrogen, total potassium, available potassium, and organic matter content in the soil (Table 1), thereby providing an extremely rich ‘soil nutrient reservoir’ for the subsequent rapeseed crop. Existing research results indicate that preceding crops with sugar beets can enhance soil microbial communities and increase the abundance of actinomycetes and streptomycetes compared to continuous cropping. The levels of available nitrogen, potassium, and soil organic matter in rotated soils are higher than those in continuously cropped soils [23,24]. This is consistent with our research findings. TaBn and SbBn also demonstrated significant effects in enhancing available potassium. Previous studies have reported that *Triticum aestivum* can alleviate soil potassium depletion, showing a relatively strong response to potassium fertilizer [25].

TaBn pattern exhibits unparalleled advantages, as it can comprehensively and significantly activate soil enzyme activities (such as S_UE, S_ACP, S_GC, and S_SC), which enhances the mineralization and cycling rates of nutrients such as nitrogen, phosphorus, and sulfur in the soil (Figure 1).

### 4.2. Response and Remodeling of Soil Microbial Community Structure and Function

Different preceding crops significantly influence the structure and function of soil microbial communities by altering soil properties, root exudates, and crop residue characteristics. Alpha diversity analysis revealed that the TaBn patterns supported the most diverse bacterial and fungal communities (Figure 3), a finding consistent with reports that Triticum aestivum enriches beneficial microbes through root exudates, thereby enhancing community diversity and functional redundancy [26]. However, this high diversity was accompanied by lower evenness (elevated Simpson index), indicating stronger dominance of certain species. In contrast, BnBn resulted in a 30% reduction in bacterial OTUs compared to TaBn and constrained fungal diversity, reflecting an imbalanced soil microecology (Figure 2). PCoA further confirmed that TaBn and BvBn systems were distinctly separated from other pattern (Figure 4).

At the phylum level, Ascomycota was the dominant fungal group across all treatment groups. Compared to continuous cropping of rapeseed, its relative abundance increased with the patterns of different crops (Figure 5), which is consistent with previous research findings [27]. The phylum Mucoromycota represents typical nutritional-type microorganisms, whose abundance has declined, potentially due to niche competition, particularly from competitors within the phylum Ascomycota [28].

Community structure analysis using PCoA and LEfSe (Figure 6) indicated that TaBn and BvBn treatments established a distinct and beneficial ecological niche, enriched with functional genera such as *Sphingomonas* (involved in C/N cycling) [29,30], *Terriglobus* [31], and the nitrogen-fixing *Bradyrhizobium* [32]. A diverse microbiome is generally more resistant to pathogen invasion [33]. Notably, under the conditions of continuous planting of BnBn, bacterial communities exhibited a lower susceptibility compared to fungal communities. Previous studies have reported that continuous sugar beet cropping leads to lower bacterial α-diversity and higher fungal α-diversity in soil microbial communities, while also increasing the abundance of potential pathogenic fungi [34,35]. This aligns with our conclusions. Furthermore, the increase in the relative abundance of fungi (including both endophytic and saprophytic groups) indicates a decline in the health status of the soil ecosystem [13,36]. Other patterns (TaBn, BvBn, GmBn, SbBn, and HvBn) shaped functionally specialized microbial communities, with functions such as residue degradation by *Wood Saprotrophs* being rotation-dependent [37].

However, FUNGuild-based functional prediction highlighted a potential risk: TaBn and BvBn treatments showed a relatively high proportion of pathogenic fungi (Figure 9), indicating that while microecological benefits are achieved, disease risk remains a concern. In comparison, GmBn patterns not only offered limited improvement in community structure but also promoted the pathogenic genus *Xanthomonas* [38], making it one of the less favorable preceding crops.

### 4.3. Key Soil Drivers and Their Association with Crop Performance

The RDA (Figure 7) and Mantel (Figure 8) tests clearly indicate that the environmental factors driving the aforementioned changes include TN, OM, and key soil enzymes (S_ACP, S_GC, and S_SC), which serve as the core driving forces regulating microbial community structure. Bacteria exhibit a stronger dependence on TN, while fungi display less sensitivity to it (Figure 8). This result is consistent with previous research, indicating that soil bacteria are more sensitive to nitrogen than fungi [39]. TN is a significant regulatory factor for bacterial communities but has no notable impact on fungi, highlighting the higher demand for nitrogen sources by bacteria (e.g., *Pseudomonas*) compared to the tolerance or alternative acquisition strategies of fungi (e.g., acquiring nitrogen through lignin decomposition) [40]. The functional differentiation among dominant microbes is evident: bacteria (such as *Pseudomonas* and *rhizobia*)focus on nitrogen cycling and high organic matter utilization, whereas fungi (e.g., *Aspergillus*) concentrate on carbon cycling (specifically carbohydrate degradation) [41]. This is consistent with previous research, indicating that preceding crops can regulate the carbon cycle through specific microbial mechanisms [42].

Together, they form a ‘functional complementary network of carbon and nitrogen cycling’ in the soil, where microbes may adjust their metabolic strategies based on resource availability [43]. This creates a positive feedback loop: excellent preceding crops (such as *Triticum aestivum* and *Beta vulgaris*) enhance soil TN, OM, and enzyme activity, which shape beneficial and diverse microbial communities, further promoting nutrient cycling and plant health, ultimately benefiting subsequent rapeseed growth. In this cycle, TaBn excels in both ‘driving factors’ (enzyme activity) and ‘response outcomes’ (microbial diversity), while BvBn demonstrates greater robustness in the fundamental driving forces (TN and OM).

### 4.4. Limitations of the Study

This study has several key limitations: single time-point sampling cannot capture temporal dynamics of soil fungal communities, lack of plant growth/yield data hinders linking microbial patterns to ecosystem functions, use of OTUs instead of ASVs masks fine-scale taxonomic variations, and the short-term trial fails to reflect long-term rotation effects. As highlighted by previous studies, FUNGuild relies on pre-defined taxon-guild associations and tends to overclassify or misclassify soil fungal taxa with unknown functions, leading to high prediction errors in soil systems [44]. Therefore, the functional profiles reported here are preliminary, and future validation using culture-dependent methods, metatranscriptomics, or metabolomics is required to confirm the actual ecological functions of the soil fungal communities.

## 5. Conclusions

Compared to continuous BnBn, the BvBn significantly increased TN, TK, AP, and OM contents. Preceding crops involving TaBn, BvBn, and SbSn generally enhanced soil S-ACP, S-β-GC, and S-SC, though none of the patterns improved S-ACPT activity. Microbial analysis revealed that the TaBn significantly increased soil microbial alpha diversity and OTU numbers. Different preceding crop plants formed distinct microbial community structures, with BvBn and TaBn showing the most unique bacterial communities and BnBn exhibiting the most distinctive fungal community. Preceding crop patterns markedly altered microbial composition at both phylum and genus levels while enriching specific indicator taxa. TN, OM, and multiple enzyme activities are key environmental factors driving microbial community evolution. Functional prediction revealed that preceding crops altered the distribution of fungal trophic functional groups, while bacteria maintained high activity in core functions such as amino acid metabolism and carbohydrate metabolism. In summary, preceding crops profoundly influences soil ecological functions by regulating soil nutrients, enzyme activities, and microbial communities. Among them, TaBn rotations demonstrate significant advantages in improving soil microbial diversity and physicochemical properties, providing scientific basis for optimizing rotation pattern selection.

## Figures and Tables

**Table 1:** The effects of different preceding crops on soil physicochemical properties.

	pH	TN (g/kg)	TP (g/kg)	TK (g/kg)	AP (mg/kg)	AK (mg/kg)	OM (%)
BnBn	6.67 ± 0.07 ab *	1.71 ± 0.01 b	1.06 ± 0.02 a	20.23 ± 0.38 b	68.38 ± 0.93 a	192.48 ± 3.56 d	5.29 ± 0.09 b
BvBn	6.68 ± 0.11 ab	1.94 ± 0.05 a	1.01 ± 0.03 a	22.35 ± 0.27 a	65.65 ± 0.5 b	245.72 ± 2.59 a	5.88 ± 0.05 a
HvBn	6.84 ± 0.03 a	1.43 ± 0.05 d	1.05 ± 0.04 a	20.25 ± 0.72 b	47.75 ± 0.8 e	194.45 ± 3.18 d	3.68 ± 0.04 e
SbBn	6.67 ± 0.03 ab	1.66 ± 0.06 b	0.91 ± 0.03 b	19.64 ± 0.41 b	64.28 ± 0.46 bc	212.22 ± 4.69 c	4.78 ± 0.04 c
TaBn	6.69 ± 0.06 ab	1.54 ± 0.04 c	0.91 ± 0.02 b	20.35 ± 0.54 b	63.37 ± 1.13 c	229.00 ± 5.27 b	4.92 ± 0.11 c
GmBn	6.56 ± 0.13 b	1.71 ± 0.03 d	0.91 ± 0.01 b	20.30 ± 0.49 b	60.50 ± 0.61 d	195.95 ± 2.24 d	4.42 ± 0.02 d

* Data were analyzed by one-way analysis of variance (one-way ANOVA) followed by Tukey’s multiple comparison test using the SPSS 26. The letters a, b, c, d and e indicate statistically significant differences, *p* < 0.05 (a > b > c > d > e).

## Data Availability

The original contributions presented in this study are included in the article. Further inquiries can be directed to the corresponding author.

## Supplementary Materials

The following supporting information can be downloaded at: https://www.mdpi.com/article/10.3390/microorganisms14010091/s1, Figure S1: Shannon diversity index dilution curve.
