# Supplementary figures and images for: Effects of Different Crop Rotations on Microbial Diversity and Enzyme Activities in Brassica napus Rhizosphere Soil
Source: Microorganisms. 2025 Dec 31;14(1):91. doi: 10.3390/microorganisms14010091 (PMC12844264; doi:10.3390/microorganisms14010091)

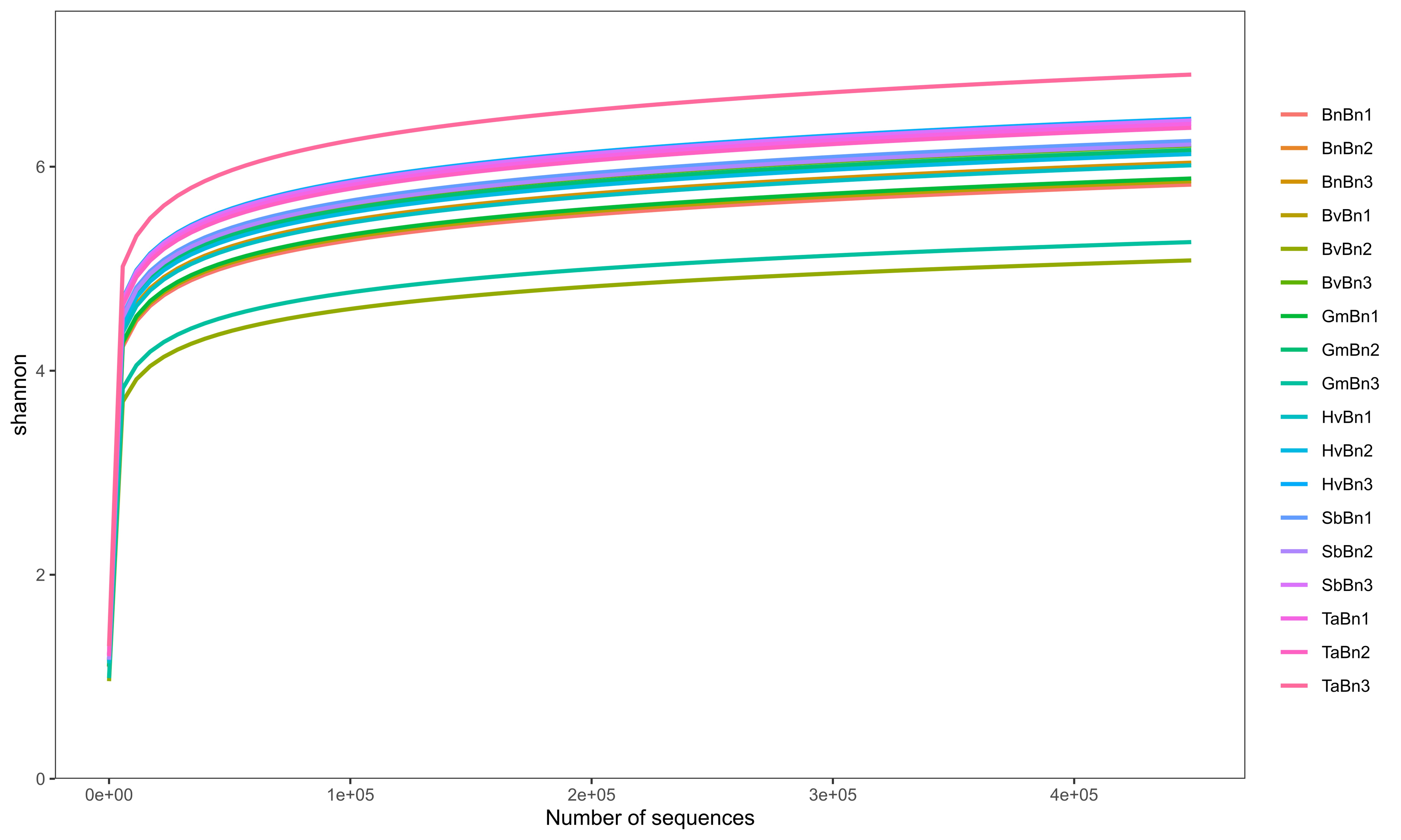

Supplement: Supplementary file 1 [file microorganisms-14-00091-s001.zip › microorganisms-4055860-supplementary.jpg]
